# Tetraphenyl­arsonium *cis*-bis­[1,2-bis­(tri­fluoro­meth­yl)ethene-1,2-dithiol­ato]platinate(II)

**DOI:** 10.1107/S1600536809021527

**Published:** 2009-06-13

**Authors:** Stephanie Hosking, Alan J. Lough, Ulrich Fekl

**Affiliations:** a University of Toronto Mississauga, Department of Chemical and Physical Sciences, 3359 Mississauga Road North, Mississauga, Ontario, Canada L5L 1C6; b X-ray Crystallography Laboratory, Department of Chemistry, University of Toronto, 80 St George St., Toronto, Ontario, Canada M5S 3H6

## Abstract

In the title compound, (C_24_H_20_As)[Pt(C_4_F_6_S_2_)_2_], the cation lies on a twofold rotation axis while the anion has crystallographic inversion symmetry. The Pt^II^ ion is in a slightly distorted square-planar coordination environment. The F atoms of both unique –CF_3_ groups are disordered over two sets of sites, the ratios of refined occupancies being 0.677 (15):0.323 (15) and 0.640 (16):0.360 (16). The crystal structure is the first to date of a monoanionic [Pt(tfd)_2_]^−^ species [tfd is 1,2-bis­(trifluoro­meth­yl)ethene-1,2-dithiol­ate] with a non-redox-active cation.

## Related literature

For background information, see: Ray *et al.* (2005 ▶); Wang & Stiefel (2001 ▶); Harrison *et al.* (2006 ▶). For related crystal structures, see: Kogut *et al.* (2006 ▶); Tang *et al.* (2009 ▶); Kasper & Inter­rante (1976 ▶); Lim *et al.* (2001 ▶). For synthetic details, see: Davison *et al.* (1964 ▶). For the treatment of disordered solvent of crystallization, see: Spek (2009 ▶); Stähler *et al.* (2001 ▶); Cox *et al.* (2003 ▶); Mohamed *et al.* (2003 ▶); Athimoolam *et al.* (2005 ▶). For a detailed description of the electronic structure of metal–dithiol­ene complexes, see: Kirk *et al.* (2004 ▶).
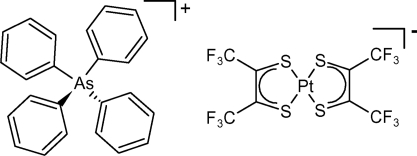


## Experimental

### 

#### Crystal data

(C_24_H_20_As)[Pt(C_4_F_6_S_2_)_2_]
*M* *_r_* = 1030.76
Monoclinic, 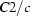

*a* = 24.9649 (10) Å
*b* = 7.3189 (3) Å
*c* = 23.6773 (6) Å
β = 117.779 (2)°
*V* = 3827.6 (2) Å^3^
*Z* = 4
Mo *K*α radiation
μ = 4.82 mm^−1^
*T* = 150 K
0.24 × 0.21 × 0.16 mm

#### Data collection

Nonius KappaCCD diffractometer
Absorption correction: multi-scan (*SORTAV*; Blessing, 1995 ▶) *T*_min_ = 0.341, *T*_max_ = 0.471
13097 measured reflections
4282 independent reflections
3269 reflections with *I* > 2σ(*I*)
*R*_int_ = 0.051

#### Refinement

*R*[*F* ^2^ > 2σ(*F* ^2^)] = 0.054
*wR*(*F* ^2^) = 0.156
*S* = 1.09
4282 reflections
230 parameters
60 restraints
H-atom parameters constrained
Δρ_max_ = 4.18 e Å^−3^
Δρ_min_ = −2.83 e Å^−3^

### 

Data collection: *COLLECT* (Nonius, 2002 ▶); cell refinement: *DENZO–SMN* (Otwinowski & Minor, 1997 ▶); data reduction: *DENZO–SMN*; program(s) used to solve structure: *SHELXS97* (Sheldrick, 2008 ▶); program(s) used to refine structure: *SHELXL97* (Sheldrick, 2008 ▶); molecular graphics: *ORTEP-3* (Farrugia, 1997 ▶); software used to prepare material for publication: *SHELXTL* (Sheldrick, 2008 ▶).

## Supplementary Material

Crystal structure: contains datablocks global, I. DOI: 10.1107/S1600536809021527/su2119sup1.cif

Structure factors: contains datablocks I. DOI: 10.1107/S1600536809021527/su2119Isup2.hkl

Additional supplementary materials:  crystallographic information; 3D view; checkCIF report

## Figures and Tables

**Fig. 1.**
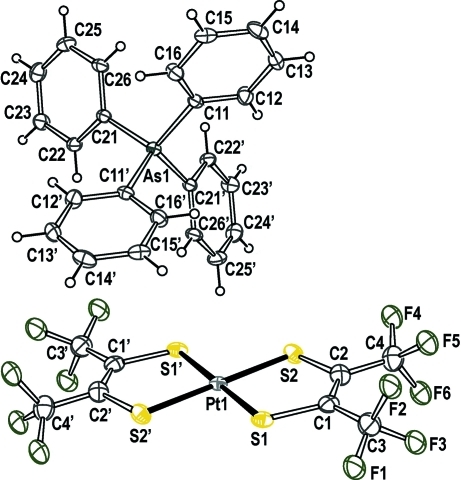
A view of the molecular structure of the title compound. Displacement ellipsoids are drawn at the 30% probabilty level. For the cation and anion, the primed labels are related by the symmetry operators (-x, y, -z + 1/2) and (-x + 1/2, -y + 1/2, -z + 1), respectively.

**Fig. 2.**
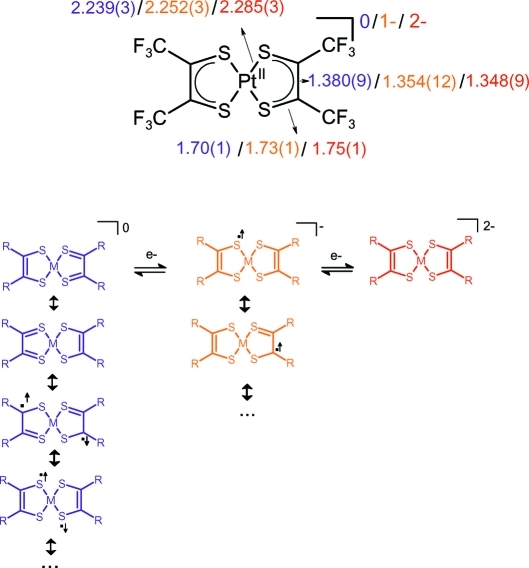
Comparison of the structural features of [Pt(tfd)2]0 (Kogut et al.,2006), [Pt(tfd)2]- (this work) and [Pt(tfd)2]2- (Tang et al., 2009).

## supplementary crystallographic information

### Comment

Square-planar metal bisdithiolene compounds are of fundamental importance for our understanding of metal complexes containing non-innocent ligands (Ray *et al.*, 2005). The nickel complex [Ni(tfd)_2_] (tfd = S_2_C_2_(CF_3_)_2_) has received considerable attention due to its potential applicability in ethylene separation and purification (Wang & Stiefel, 2001). The one-electron reduced form of the metal complex is involved in the mechanism of alkene binding (Harrison *et al.*, 2006). In the course of our studies on the analogous platinum complexes we have crystallographically characterized [Pt(tfd)_2_]^0^ and [Pt(tfd)_2_]^2-^ (Kogut *et al., *2006; Tang *et al.*, 2009, respectively). These complexes contain square-planar platinum(II), and the tfd ligand is redox-active. While tfd is not fully reduced in the charge-neutral complex (and is only formally 1,2-perfluoromethylethene-1,2-dithiolate), tfd is fully reduced in the dianion. The C—C bond in the chelate ring of the neutral complex shortens upon reduction (Tang *et al.*, 2009).

We report here on the first crystal structure of monoanionic [Pt(tfd)_2_]^-^ with a non-redox-active cation (Fig. 1). A report on a previous structural determination (Kasper & Interrante, 1976) of the charge-transfer complex between tetrathiofulvalene and charge-neutral [Pt(tfd)_2_] has proposed that [Pt(tfd)_2_] is reduced by one electron and tetrathiovulvalene is oxidized to tetrathiafulvalinium; however, in order to reliably establish the structural properties of the one-electron-reduced species, use of a non-redox-active cation is preferable. [Pt(tfd)_2_] in the title compound reported here is clearly monoanionic. The structure reported here completes structural characterization of the redox series [Pt(tfd)_2_]^0/1-/2-^. Structural features of [Pt(tfd)_2_]^-^ (Fig. 2) are intermediate with respect to those of [Pt(tfd)_2_]^0^ (Kogut *et al.*, 2006) and [Pt(tfd)_2_]^2-^ (Tang *et al.*, 2009). The structural effects observed upon stepwise reduction of the neutral complex [Pt(tfd)_2_]^0^ to [Pt(tfd)_2_]^-^ and [Pt(tfd)_2_]^2-^ are of varying statistical significance (significant within 2σ for Pt—S, borderline significant for C—C). However, they confirm observations made by Lim *et al.* (2001) on an analogous nickel complex containing a non-fluorinated dithiolene, the redox series [Ni(S_2_C_2_Me_2_)_2_]^0,1-,2-^. The combined evidence suggests that these effects are real. A relatively straightforward rationalization, in terms of resonance structures, is shown in Fig. 2. For a detailed description of the electronic structure of metal dithiolene complexes, see Kirk *et al.* (2004).

### Experimental

The tetraphenylarsonium salt of [Pt(tfd)_2_]^-^ was synthesized using a slightly modified literature procedure. The literature procedure uses acetone/ethanol as the reductant, followed by precipitation with tetraphenylarsonium chloride, (Davison *et al.*, 1964) to obtain the product in 36% yield. We repeated that literature reaction and obtained 24% yield. We then decided to use water in THF to achieve reduction of [Pt(tfd)_2_], followed by addition of tetraphenylarsonium chloride. It was previously observed that water in polar solvents such as THF leads to reduction of the related nickel complex Ni[(S_2_C_2_(CF_3_)_2_]_2_ (Harrison *et al.*, 2006). In a 10 ml vial, 24.9 mg (0.0385 mmol) of [Pt(tfd)_2_] were dissolved in 1.0 ml ofTHF, leading to a dark greenish-blue solution. Addition of 0.3 ml of H_2_O induced a rapid colour change, to produce a brownish-yellow solution. Solid tetraphenylarsonium chloride (25.3 mg, 0.068 mmol) was added to the solution while stirring, followed by an additional 0.25 ml of THF, to produce a homogeneous solution. In order to lower the solubility of the salt again, an additional 0.4 ml of H_2_O were added, and the vial was storred at 278 K for three weeks. Crystals had not formed at that time, and crystallization was induced by scratching of the inner surface of the vial with a glass rod. After three additional weeks at 378 K, [AsPh_4_]^+^[Pt(tfd)_2_]^-^ had crystallized as dark yellow blocks. The crystals were removed from the supernatant and dried under vacuum. Yield: 26.6 mg (0.0258 mmol, 67%). Inspections of the crystals with a stereomicroscope showed them to be of excellent quality. One crystal was chosen for the single-crystal X-ray structure determination.

### Refinement

H atoms were placed in calculated positions and treated as riding: C—H = 0.95 Å with *U*_iso_(H) = 1.2*U*_iso_(C). The F atoms of both unique –CF_3_ groups are disordered over two sets of sites with the ratio of refined occupancies being 0.677 (15):0.323 (15) for F1/F2/F3:F1A/F2A/F3A and 0.640 (16):0.360 (16) for F4/F5/F6:F4A/F5A/F6A. The SADI and EADP commands in the *SHELXTL* (Sheldrick, 2008) software were used to restrain the parameters of the disordered groups. During the refinement of the structure, electron density peaks were located that were believed to be highly disordered solvent molecules (possibly THF). Attempts made to model the solvent molecule were not successful. The SQUEEZE option in PLATON (Spek, 2009) indicated there was a solvent cavity of volume 130.0 Å^3^ containing approximately 18 electrons. In the final cycles of refinement, this contribution to the electron density was removed from the observed data. The density, the F(000) value, the molecular weight and the formula are given without taking into account the results obtained with the SQUEEZE option PLATON (Spek, 2009). Similar treatments of disordered solvent molecules have been carried out in this manner (Stähler *et al.*,  2001; Cox *et al.*,  2003; Mohamed *et al.*,  2003; Athimoolam *et al.*,  2005).

### Crystal data

**Table:**

(C_24_H_20_As)[Pt(C_4_F_6_S_2_)_2_]	*F*(000) = 1980
*M**_r_* = 1030.76	*D*_x_ = 1.789 Mg m^−^^3^
Monoclinic, *C*2/*c*	Mo *K*α radiation, λ = 0.71073 Å
Hall symbol: -C 2yc	Cell parameters from 13097 reflections
*a* = 24.9649 (10) Å	θ = 2.9–27.5°
*b* = 7.3189 (3) Å	µ = 4.82 mm^−^^1^
*c* = 23.6773 (6) Å	*T* = 150 K
β = 117.779 (2)°	Block, orange
*V* = 3827.6 (2) Å^3^	0.24 × 0.21 × 0.16 mm
*Z* = 4	

### Data collection

**Table:**

Nonius KappaCCD diffractometer	4282 independent reflections
Radiation source: fine-focus sealed tube	3269 reflections with *I* > 2σ(*I*)
graphite	*R*_int_ = 0.051
Detector resolution: 9 pixels mm^-1^	θ_max_ = 27.5°, θ_min_ = 2.9°
φ scans and ω scans with κ offsets	*h* = −32→25
Absorption correction: multi-scan (*SORTAV*; Blessing, 1995)	*k* = −8→9
*T*_min_ = 0.341, *T*_max_ = 0.471	*l* = −25→30
13097 measured reflections	

### Refinement

**Table:**

Refinement on *F*^2^	Primary atom site location: structure-invariant direct methods
Least-squares matrix: full	Secondary atom site location: difference Fourier map
*R*[*F*^2^ > 2σ(*F*^2^)] = 0.054	Hydrogen site location: inferred from neighbouring sites
*wR*(*F*^2^) = 0.156	H-atom parameters constrained
*S* = 1.09	*w* = 1/[σ^2^(*F*_o_^2^) + (0.0976*P*)^2^ + 3.8529*P*] where *P* = (*F*_o_^2^ + 2*F*_c_^2^)/3
4282 reflections	(Δ/σ)_max_ < 0.001
230 parameters	Δρ_max_ = 4.18 e Å^−^^3^
60 restraints	Δρ_min_ = −2.83 e Å^−^^3^

### Special details

**Table:**

Geometry. All e.s.d.'s (except the e.s.d. in the dihedral angle between two l.s. planes) are estimated using the full covariance matrix. The cell e.s.d.'s are taken into account individually in the estimation of e.s.d.'s in distances, angles and torsion angles; correlations between e.s.d.'s in cell parameters are only used when they are defined by crystal symmetry. An approximate (isotropic) treatment of cell e.s.d.'s is used for estimating e.s.d.'s involving l.s. planes.
Refinement. Refinement of *F*^2^ against ALL reflections. The weighted *R*-factor *wR* and goodness of fit *S* are based on *F*^2^, conventional *R*-factors *R* are based on *F*, with *F* set to zero for negative *F*^2^. The threshold expression of *F*^2^ > σ(*F*^2^) is used only for calculating *R*-factors(gt) *etc*. and is not relevant to the choice of reflections for refinement. *R*-factors based on *F*^2^ are statistically about twice as large as those based on *F*, and *R*- factors based on ALL data will be even larger.

### Fractional atomic coordinates and isotropic or equivalent isotropic displacement parameters (Å^2^)

**Table:**

	*x*	*y*	*z*	*U*_iso_*/*U*_eq_	Occ. (<1)
Pt1	0.2500	0.2500	0.5000	0.02912 (17)	
S1	0.22659 (8)	0.5216 (3)	0.44973 (9)	0.0371 (4)	
S2	0.30913 (9)	0.1915 (3)	0.45362 (10)	0.0408 (5)	
F1	0.2074 (3)	0.7827 (10)	0.3529 (5)	0.061 (2)	0.676 (15)
F2	0.3033 (3)	0.8005 (11)	0.3897 (5)	0.061 (2)	0.676 (15)
F3	0.2523 (3)	0.6463 (10)	0.3070 (3)	0.061 (2)	0.676 (15)
F1A	0.2129 (6)	0.680 (3)	0.3037 (4)	0.078 (7)*	0.324 (15)
F2A	0.2298 (8)	0.795 (2)	0.3910 (9)	0.071 (6)*	0.324 (15)
F3A	0.2988 (6)	0.796 (2)	0.3648 (8)	0.061 (6)*	0.324 (15)
F4	0.3847 (4)	0.2453 (10)	0.3995 (4)	0.062 (2)	0.638 (16)
F5	0.3645 (4)	0.5234 (10)	0.3690 (4)	0.062 (2)	0.638 (16)
F6	0.3084 (3)	0.3029 (13)	0.3109 (3)	0.062 (2)	0.638 (16)
F6A	0.2985 (6)	0.440 (3)	0.3141 (6)	0.092 (7)*	0.362 (16)
F4A	0.3588 (9)	0.212 (2)	0.3578 (12)	0.137 (12)*	0.362 (16)
F5A	0.3837 (5)	0.4871 (18)	0.3956 (7)	0.068 (6)*	0.362 (16)
C1	0.2649 (3)	0.5226 (11)	0.4045 (3)	0.0394 (17)	
C2	0.3007 (4)	0.3803 (12)	0.4073 (4)	0.0438 (19)	
C3	0.2566 (3)	0.6806 (13)	0.3656 (3)	0.059 (2)	
C4	0.3370 (3)	0.3656 (8)	0.3723 (3)	0.059 (2)	
As1	0.0000	0.61646 (13)	0.2500	0.0248 (2)	
C11	0.0654 (3)	0.4612 (9)	0.2596 (3)	0.0261 (13)	
C12	0.0908 (3)	0.4759 (10)	0.2191 (3)	0.0367 (16)	
H12A	0.0767	0.5658	0.1864	0.044*	
C13	0.1372 (3)	0.3578 (12)	0.2266 (4)	0.0415 (19)	
H13A	0.1550	0.3674	0.1989	0.050*	
C14	0.1573 (4)	0.2281 (10)	0.2733 (5)	0.041 (2)	
H14A	0.1892	0.1483	0.2781	0.049*	
C15	0.1313 (4)	0.2115 (11)	0.3144 (4)	0.0393 (19)	
H15A	0.1455	0.1211	0.3469	0.047*	
C16	0.0849 (3)	0.3278 (11)	0.3071 (3)	0.0348 (16)	
H16A	0.0665	0.3170	0.3341	0.042*	
C21	−0.0245 (3)	0.7717 (8)	0.1779 (3)	0.0254 (14)	
C22	0.0166 (3)	0.9043 (10)	0.1792 (3)	0.0302 (15)	
H22A	0.0563	0.9104	0.2141	0.036*	
C23	−0.0014 (3)	1.0262 (10)	0.1288 (3)	0.0354 (16)	
H23A	0.0260	1.1159	0.1286	0.042*	
C24	−0.0600 (4)	1.0162 (11)	0.0784 (4)	0.0425 (18)	
H24A	−0.0722	1.0999	0.0439	0.051*	
C25	−0.1002 (3)	0.8881 (12)	0.0776 (3)	0.0426 (19)	
H25A	−0.1401	0.8838	0.0430	0.051*	
C26	−0.0826 (3)	0.7638 (9)	0.1277 (4)	0.0333 (17)	
H26A	−0.1103	0.6744	0.1274	0.040*	

### Atomic displacement parameters (Å^2^)

**Table:**

	*U*^11^	*U*^22^	*U*^33^	*U*^12^	*U*^13^	*U*^23^
Pt1	0.0227 (2)	0.0289 (2)	0.0304 (2)	0.00284 (14)	0.00796 (18)	−0.00095 (14)
S1	0.0276 (9)	0.0297 (10)	0.0446 (10)	0.0037 (7)	0.0090 (8)	0.0038 (8)
S2	0.0384 (11)	0.0366 (10)	0.0520 (12)	0.0004 (9)	0.0248 (9)	−0.0049 (9)
F1	0.053 (3)	0.061 (3)	0.075 (4)	0.007 (2)	0.035 (3)	0.029 (3)
F2	0.053 (3)	0.061 (3)	0.075 (4)	0.007 (2)	0.035 (3)	0.029 (3)
F3	0.053 (3)	0.061 (3)	0.075 (4)	0.007 (2)	0.035 (3)	0.029 (3)
F4	0.056 (4)	0.080 (5)	0.066 (4)	0.015 (3)	0.042 (3)	0.001 (3)
F5	0.056 (4)	0.080 (5)	0.066 (4)	0.015 (3)	0.042 (3)	0.001 (3)
F6	0.056 (4)	0.080 (5)	0.066 (4)	0.015 (3)	0.042 (3)	0.001 (3)
C1	0.034 (4)	0.036 (4)	0.037 (4)	−0.014 (3)	0.007 (3)	−0.001 (3)
C2	0.047 (4)	0.040 (5)	0.045 (4)	−0.019 (4)	0.021 (4)	−0.014 (4)
C3	0.056 (6)	0.062 (6)	0.056 (6)	−0.021 (5)	0.023 (5)	0.001 (5)
C4	0.073 (7)	0.055 (6)	0.056 (5)	−0.009 (5)	0.036 (5)	−0.009 (4)
As1	0.0215 (5)	0.0261 (5)	0.0256 (5)	0.000	0.0099 (4)	0.000
C11	0.020 (3)	0.026 (3)	0.031 (3)	−0.001 (3)	0.010 (3)	−0.010 (3)
C12	0.042 (4)	0.036 (4)	0.036 (4)	0.009 (3)	0.021 (3)	0.003 (3)
C13	0.035 (4)	0.055 (6)	0.042 (4)	0.008 (4)	0.025 (3)	0.002 (4)
C14	0.028 (4)	0.032 (5)	0.061 (6)	0.006 (3)	0.018 (4)	−0.010 (3)
C15	0.028 (4)	0.038 (4)	0.037 (4)	0.002 (3)	0.002 (3)	0.002 (3)
C16	0.031 (4)	0.034 (4)	0.041 (4)	0.004 (3)	0.018 (3)	0.005 (3)
C21	0.020 (3)	0.028 (4)	0.027 (3)	0.000 (2)	0.010 (3)	−0.002 (2)
C22	0.026 (3)	0.033 (4)	0.027 (3)	−0.004 (3)	0.009 (3)	0.002 (3)
C23	0.035 (4)	0.030 (4)	0.040 (4)	−0.001 (3)	0.016 (3)	0.011 (3)
C24	0.054 (5)	0.039 (5)	0.037 (4)	0.007 (4)	0.023 (4)	0.009 (3)
C25	0.031 (4)	0.054 (5)	0.031 (4)	0.008 (4)	0.004 (3)	0.009 (4)
C26	0.019 (3)	0.043 (5)	0.034 (4)	0.003 (3)	0.009 (3)	0.005 (3)

### Geometric parameters (Å, °)

**Table:**

Pt1—S1^i^	2.2496 (18)	As1—C11	1.913 (6)
Pt1—S1	2.2496 (19)	C11—C12	1.378 (9)
Pt1—S2^i^	2.254 (2)	C11—C16	1.394 (10)
Pt1—S2	2.254 (2)	C12—C13	1.390 (10)
S1—C1	1.737 (8)	C12—H12A	0.9500
S2—C2	1.715 (9)	C13—C14	1.364 (12)
F1—C3	1.346 (7)	C13—H13A	0.9500
F2—C3	1.355 (6)	C14—C15	1.402 (13)
F3—C3	1.365 (6)	C14—H14A	0.9500
F1A—C3	1.360 (7)	C15—C16	1.382 (11)
F2A—C3	1.372 (7)	C15—H15A	0.9500
F3A—C3	1.359 (7)	C16—H16A	0.9500
F4—C4	1.376 (6)	C21—C26	1.382 (10)
F5—C4	1.365 (6)	C21—C22	1.403 (9)
F6—C4	1.366 (6)	C22—C23	1.386 (10)
F6A—C4	1.371 (6)	C22—H22A	0.9500
F4A—C4	1.360 (6)	C23—C24	1.394 (11)
F5A—C4	1.362 (6)	C23—H23A	0.9500
C1—C2	1.353 (12)	C24—C25	1.368 (11)
C1—C3	1.432 (13)	C24—H24A	0.9500
C2—C4	1.489 (12)	C25—C26	1.394 (11)
As1—C21^ii^	1.899 (7)	C25—H25A	0.9500
As1—C21	1.899 (7)	C26—H26A	0.9500
As1—C11^ii^	1.913 (6)		
			
S1^i^—Pt1—S1	180.00 (10)	C21^ii^—As1—C11	110.4 (3)
S1^i^—Pt1—S2^i^	88.73 (8)	C21—As1—C11	111.2 (3)
S1—Pt1—S2^i^	91.27 (8)	C11^ii^—As1—C11	107.1 (4)
S1^i^—Pt1—S2	91.27 (8)	C12—C11—C16	120.9 (6)
S1—Pt1—S2	88.73 (8)	C12—C11—As1	121.0 (5)
S2^i^—Pt1—S2	180.000 (1)	C16—C11—As1	118.0 (5)
C1—S1—Pt1	104.3 (3)	C11—C12—C13	119.4 (7)
C2—S2—Pt1	104.2 (3)	C11—C12—H12A	120.3
C2—C1—C3	123.2 (7)	C13—C12—H12A	120.3
C2—C1—S1	120.5 (6)	C14—C13—C12	120.3 (7)
C3—C1—S1	116.3 (6)	C14—C13—H13A	119.8
C1—C2—C4	126.0 (7)	C12—C13—H13A	119.8
C1—C2—S2	122.1 (6)	C13—C14—C15	120.6 (7)
C4—C2—S2	111.9 (6)	C13—C14—H14A	119.7
F1—C3—F2	104.5 (7)	C15—C14—H14A	119.7
F3A—C3—F1A	102.9 (8)	C16—C15—C14	119.4 (8)
F1—C3—F3	104.1 (7)	C16—C15—H15A	120.3
F2—C3—F3	101.0 (6)	C14—C15—H15A	120.3
F3A—C3—F2A	99.4 (7)	C15—C16—C11	119.3 (7)
F1A—C3—F2A	99.5 (7)	C15—C16—H16A	120.3
F1—C3—C1	115.9 (7)	C11—C16—H16A	120.3
F2—C3—C1	114.5 (7)	C26—C21—C22	120.8 (6)
F1A—C3—C1	119.6 (10)	C26—C21—As1	121.1 (5)
F3—C3—C1	115.2 (7)	C22—C21—As1	117.9 (5)
F2A—C3—C1	99.6 (11)	C23—C22—C21	119.1 (6)
F4A—C4—F5A	105.9 (8)	C23—C22—H22A	120.5
F5—C4—F6	104.5 (6)	C21—C22—H22A	120.5
F4A—C4—F6A	104.3 (8)	C22—C23—C24	119.5 (7)
F5A—C4—F6A	102.4 (7)	C22—C23—H23A	120.2
F5—C4—F4	102.7 (6)	C24—C23—H23A	120.2
F6—C4—F4	101.1 (6)	C25—C24—C23	121.3 (7)
F4A—C4—C2	128.2 (13)	C25—C24—H24A	119.4
F5A—C4—C2	110.8 (9)	C23—C24—H24A	119.4
F5—C4—C2	115.0 (6)	C24—C25—C26	119.8 (7)
F6—C4—C2	117.8 (7)	C24—C25—H25A	120.1
F6A—C4—C2	102.1 (10)	C26—C25—H25A	120.1
F4—C4—C2	113.8 (7)	C21—C26—C25	119.5 (7)
C21^ii^—As1—C21	106.5 (4)	C21—C26—H26A	120.2
C21^ii^—As1—C11^ii^	111.2 (3)	C25—C26—H26A	120.2
C21—As1—C11^ii^	110.4 (3)		

Symmetry codes: (i) −*x*+1/2, −*y*+1/2, −*z*+1; (ii) −*x*, *y*, −*z*+1/2.
